# Gambling in Spanish Adolescents: Prevalence and Association with Mental Health Indicators

**DOI:** 10.3390/ijerph19010129

**Published:** 2021-12-23

**Authors:** Alicia Pérez-Albéniz, Mario Gil, Adriana Díez-Gómez, Gema Martín-Seoane, Beatriz Lucas-Molina

**Affiliations:** 1 Department of Educational Sciences, University of La Rioja, 26002 Logrono, Spain; mgil1997@gmail.com (M.G.); adriana.diez@unirioja.es (A.D.-G.); 2 Department of Research and Psychology Education, Complutense University of Madrid, 28223 Pozuelo de Alarcon, Spain; gema.martin@psi.ucm.es; 3 Department of Developmental and Educational Psychology, University of Valencia, 46010 Valencia, Spain

**Keywords:** gambling, mental health, prevalence, adolescence, behavioral addictions

## Abstract

Concern about the development of behavioral addictions in adolescence, including gambling, has increased in recent years. Evidence shows that problem gambling can lead to personal, social, or health problems. However, even though gambling is an illegal activity, studies on this problem are quite limited in Spain. The main objective of this study was to analyze the prevalence of gambling in adolescents in Spain. Moreover, gambling behaviors were examined according to gender and age, and their possible relationship with several mental health indicators was analyzed. The results showed that 20.6% of the adolescents who participated in the study had gambled money in the past year. The highest gambling prevalence was found in boys and in adolescents from the age of 16 years old. Moreover, the results showed that gambling behavior was related to different mental health indicators.

## 1. Introduction

Although the population is more or less familiar with addiction to drugs or other substances, non-substance addiction has become a new and growing problem in modern society. These are the so-called behavioral addictions, which involve reward circuits in the brain similar to those involved in substance addictions [1].

Behavioral addictions are increasingly included as one of the types of addictions. The report by the European School Project on Alcohol and Other Drugs (ESPAD), one of the most relevant surveys in Europe on adolescent drug use, expanded its scope in 2015 and included behavioral addictions such as problematic Internet use and gambling [2]. In fact, due to the growing recognition and interest in behavioral addictions, they have been included in the most recent version of the ICD as psychological problems [3].

Some studies agree that an increase in the development of these types of addictions is taking place in society. In Spain, gambling and games of chance are widespread, and this is demonstrated by the data from the National Drug Plan [4] in the Survey on Drug use in Secondary Schools in Spain (Encuesta Sobre el Uso de Drogas en Enseñanzas Secundarias en España, ESTUDES) survey. These data reveal that, in 2017, 60.2% of the population between 15 and 64 years old had gambled with money in the past year. Along the same lines, the results of the ESPAD [2] report indicated that 14% of European students specified that they had gambled at some time, and 7% said they had done so frequently (two or more times a month) in the past 12 months. The latest European report [5] revealed an increase in the prevalence of gambling with money in the past 12 months, given that 22% of European students from 15–16 years old declared that they had gambled with money (online, in person or both).

Addictive behaviors, with or without substances, generally appear in adolescence or adulthood, and they are more frequent in these age groups than in any other [6]. Adolescence is considered to be a particularly important development period for risk-taking behaviors. Authors such as Arnott and Alain [7] point out that, according to the emerging neurobiological model of addiction, neurodevelopmental changes that occur during adolescence lead to an imbalance between emotional (reward motivation) and cognitive (executive control) processes. Consequently, adolescence may represent a vulnerable period for the development of gambling problems. Therefore, it is not unreasonable to assert that this neurobiological fragility contributes to an increased risk of developing addictive behaviors in adolescence [8]. In addition, authors such as Brezing et al. [9], in relation to non-substance addictions, point to the importance of environmental factors, such as early exposure to traumatic events, a family history of addictive disorders, access to gambling, or peer influence.

The explanation for the behaviors that initiate and maintain gambling involves many factors, as is the case with most adolescent risky behaviors. For example, some risk factors are impulsivity [10,11], sensation-seeking [12], difficulty regulating emotions [13,14], alcohol and tobacco use [10,15], cannabis or illicit drug use [16], and poor academic performance [17], among others. However, one risk factor that stands out from the rest is the male gender, because it has been shown that most pathological gamblers are men [15].

Another factor that has been associated with gambling behavior is health. Studies such as the one by Okunna et al. [15] argue that an optimal state of health leads to a lower rate of gambling. Furthermore, the authors conclude that the number of days spent on physical activity could also determine addictive gambling behaviors. Social relationships, whether with family or friends, make up one of the most relevant factors associated with addictive behaviors. Caldeira et al. [18] state in their study that poor parental supervision is a risk factor for gambling. Although the family relationship seems to be a risk factor, some authors, such as Dussault et al. [19], claim that a strong affective bond with parents is a protective factor against behavioral addictions because it allows the adolescent to develop positive views about himself or herself and others. The relationship with the peer group may also be a protective factor against gambling. Bukowski et al. [20] state that adolescents who have a good relationship with a best friend are less likely to develop behavioral addictions. 

Taking into account the current gambling situation in society, especially among the adolescent population, the first objective of this study was to examine the prevalence of gambling behaviors in a representative sample of Spanish adolescents aged between 14 and 19 years. We expected the prevalence of gambling behaviors among Spanish adolescents to be similar than previous national and international studies. The second objective was to analyze the expression of gambling behaviors depending on the gender and age of the adolescent sample. We expected that men and older students would participate more frequently in gambling behaviors. The third objective was to explore the possible relationship between several mental health indicators and gambling. The specific aim was to analyze whether there was a relationship between gambling and indicators of emotional and behavioral problems, self-esteem, depression, and subjective wellbeing. We expected that those students who participated in gambling behaviors would also show worse mental health indicators. Through this study, the current situation of gambling among a sample of adolescents in Spain will be characterized. This information will allow the design and implementation of contextualized prevention programs.

## 2. Materials and Methods

### 2.1. Participants

The sample consisted of 1790 students, 816 male (45.6%) and 961 female (53.7%). Thirteen participants (0.7%) defined themselves as intersexual, transgender, or another sexual condition. The mean age was 15.70 years (SD = 1.261), and the age range was between 14 and 19 years. The nationalities of the participants were the following: 89.4% Spanish, 2.5% Romanian, 1.9% Latin American, 1.4% Moroccan, 0.8% Pakistani, 0.3% Portuguese, and 3.8% other nationalities.

To guarantee the representativeness of the sample, random stratified cluster sampling was carried out at the classroom level in a population of approximately 15,000 students in the Autonomous Community of La Rioja, a region located in northern Spain. The strata were created according to the type of school (public or private-subsidized), the academic stage (Secondary and Vocational Training Schools) and to different socio-economic levels, where the probability of extraction from the classroom was determined by the number of students (sample probability = 0.5; sample error = 5%). Thirty schools participated in the study. 

### 2.2. Instruments

1. Gambling frequency. The question on the ESTUDES survey was used. It contains two items (online and in person) that evaluate gambling frequency (number of times) on a 5-point Likert scale *(never having gambled, not having gambled in the past 12 months, gambled monthly, weekly, or daily)*.
2. Type of game in which they have bet money. The analysis was performed by using an adaptation of the 14-question instrument from the ESTUDES survey, which evaluates different types of gambling. For the two items (online and in person), the participant is asked to indicate the types of gambling in which they have participated in the past 12 months. The 12 options (multiple choice) are *Lotteries, Instant lotteries, Football pools, Sport bets, Horse racing bets, Slots and slot machines, Cash games and cards, Bingo, Video games, eSports or electronic sports, Casino games, and Games in gambling halls*.
3. Gambling intensity. An adaptation of items from the ESTUDES survey was also used. For the two items (online and in person), the questionnaire includes 5 of the 7 questions from the original questionnaire to evaluate the participants who had gambled money in the past 12 months. The five questions are associated with five categories of intensity, corresponding to different maximum amounts of money gambled in a day. The response options were: *less than €6, between €6 and €30, between €31 and €60, between €61 and €300, more than €300, I have not bet money in the past 12 months, and I have never bet money*.
4. Strengths and Difficulties Questionnaire (SDQ) [21]. The SDQ is a widely-used measurement instrument to assess behavioral and emotional difficulties and social skills. In addition, this questionnaire is commonly used as a tool for psychopathological screening and epidemiological analysis of the state of mental health in the youth population. The SDQ, through a brief, simple, and easy to administer questionnaire, makes it possible to obtain reliable samples of conducts related to emotional and behavioral problems (Fonseca, 2017). It is composed of 25 items with a 3-point Likert scale (0 = *no, never*, 1 = *sometimes*, 2 = *yes, always*). The items are grouped in five scales with five items each: Emotional Difficulties; Conduct Problems; Hyperactivity Difficulties; Problems with Peers; and Prosocial Behavior. The first four scales make up the Total Difficulties score. The higher the score, the higher the level of emotional and behavioral difficulties. In contrast, in the case of the Prosocial Behavior scale, a lower score is associated with worse behavioral adjustment [22]. In this study, we used the Spanish version of the scale [23]. The level of internal consistency of the Total difficulties score was 0.84, ranging between 0.71 and 0.75 for the SDQ subscales.
5. Rosenberg Self-esteem Scale [24]. It is a unidimensional scale that makes it possible to assess self-esteem. Its 10 items are statements that can be used to assess self-esteem (e.g., *I think I have reasons to feel proud*). The items have a 4-point Likert scale (from 1 = *strongly disagree* to 4 = *strongly agree*). The scale showed good reliability in this sample (α = 0.87).
6. Reynolds Adolescent Depression Scale Short Form (RADS-SF) [25]. This instrument, through its 10 items, evaluates the severity of depressive symptomatology in adolescence. The items cover different dimensions (Anhedonia, Somatic complaints, Negative self-evaluation. and Dysphoria), and responses are given on a 4-point Likert scale (from 1 = *almost never* to 4 = *almost always*). It is a widely used scale, validated by studies such as the one by Ortuño-Sierra et al. [26].
7. Personal Wellbeing Index-School Children (PWI-SC) [27,28]. The PWI-SC is an instrument used to assess subjective wellbeing in school-aged children and adolescents (PWI-SC). It is composed of eight items that measure, in a generic and abstract way, subjective satisfaction with a specific life domain. The items have several response options, ranging from *very dissatisfied* (score of 0) to *very satisfied* (score of 10). The first item analyzes "life as a whole", whereas the other seven items refer to satisfaction with: health; standard of living; accomplishments in life; how secure they feel; the groups of people they are part of; future security; and their relationships with other people. The overall score is obtained by adding up the scores on the seven items (except item one). The PWI-SC has been validated in previous studies with adolescents, both nationally and internationally [22]. The scale showed good reliability in this sample (α = 0.94).

### 2.3. Procedure

This research has the approval of the General Directorate of Education of the Rioja Government and the La Rioja Ethics Committee on Clinical Research (CEICLAR) in Spain. The process of contacting the schools to propose the testing was carried out by telephone and email. The first contact with the school was made through the school principal.

In order to standardize the administration of the tests, all the researchers followed the same protocol. Test administration took place collectively in groups of between 10 and 30 adolescents, using a computer, in a classroom equipped for this purpose and always during school hours.

The participants were informed of the confidentiality of their responses and the voluntary nature of their participation in the study. Likewise, informed consent was requested from the adolescents’ legal guardians for their participation in the study. The lack of informed consent was a cause of exclusion, as well as an age above 19 years. The study was presented to the participants as a study about emotional well-being and mental health.

### 2.4. Data Analysis

First, the prevalence of gambling in the past 12 months was calculated for the whole sample. We also calculated the prevalence of gambling frequency according to the modality (online and in person) and the type of gambling. 

Next, analyses of the prevalence of gambling in the past 12 months according to gender and age were performed, and possible differences based on these variables were examined using the chi-square statistic.

Second, the age of onset of gambling was analyzed. Descriptive statistics were calculated for this variable, and possible differences based on gender and age were examined using univariate analysis of variance (ANOVA).

To analyze the relationship between the different mental health indicators and having gambled money in the past 12 months, a Multivariate Analysis of Variance (MANOVA) was carried out. In the MANOVA, the variable “having gambled in the past 12 months” was considered a fixed factor, whereas the scale scores were considered dependent variables. In this study, the conventional statistical significance value (*p* < 0.05) was used. To test for significant differences between the dependent variables together, Wilks’ Lambda value was used. To determine the effect size and analyze the practical significance of the results, the partial eta squared statistic (partial *η*^2^) was used. When *η*^2^ > 0.15, the effect is large in magnitude, and when *η*^2^ < 0.06, the effect size is medium.

Because the assessment was completed via an online platform and in a supervised context (in computer classrooms during school hours under the supervision of a researcher), there were no missing data.

The data analysis was carried out with the SPSS v26 (IBM Corp, Armonk, NY, USA) statistical program [29].

## 3. Results

### 3.1. Prevalence of Gambling: Gender and Age Differences 

First, the prevalence of gambling in the past 12 months was analyzed in the whole sample. Table 1 shows that 20.6% of the sample reported having gambled money in the past 12 months.

The second question analyzed was the prevalence of in-person and online gambling in the past year. Table 1 shows that the percentage of those who said they had gambled money in person was more than twice as high (24.9%) as for those who had gambled online in the past 12 months (10.2%).

For the next analysis, with the participants who claimed to have gambled money in the past 12 months, a study was carried out of the frequency of gambling in each modality. As Table 2 shows, sports betting was the most prevalent type of gambling in both in-person and in online gambling. In the case of in-person gambling, card games with money and football pools were highly prevalent. In online gambling, video games and football pools were also noteworthy. 

Next, gambling frequencies were analyzed according to gender. As Table 1 reveals, boys said they gambled more often than girls, both in person [39.5% boys and 12.8% girls; χ^2^ (1, 1777) = 146.930, *p* < 0.05] and online [18.9% boys and 2.9% girls; χ^2^ (1, 1777) = 83.128, *p* < 0.05]. 

**Figure 1:**
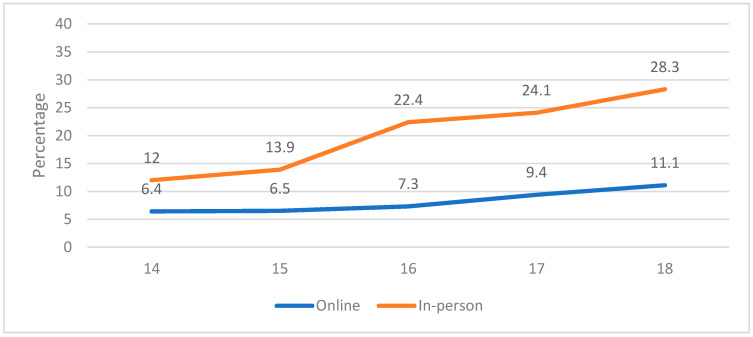
Gambling frequency by modality and age.

Gambling frequency in the past 12 months was also analyzed according to age (see Figure 1). The results show a progressive increase in gambling frequency with age in the two gambling modalities. However, this increase was not statistically significant in the online modality [χ^2^ (4, 1790) = 6.447, *p* > 0.05], and it only reached statistical significance in the in-person modality [χ^2^ (4, 1790) = 39.759, *p* < 0.05]. Specifically, the post hoc analyses showed a statistically significant increase in in-person gambling at 16 years old that was maintained until the age of 18.

Table 3 shows the mean ages of initiation in gambling games, according to the gambling modality and gender. The corresponding ANOVAs did not yield statistically significant differences based on gender in the in-person [F (1,386) = 1.966, *p* > 0.05] or online [F (1,127) = 0.017, *p* > 0.05] modality. Consequently, the age of 14 is the most frequent age of initiation in gambling for both genders and both forms of gambling.

The amount of money bet in a single day (gambling intensity) was the topic of the next analysis. The prevalence of each of the different categories of gambling intensity was calculated in the participants who claimed to have gambled money in the past 12 months. As Table 4 shows, the highest prevalence for both in-person and online gambling corresponds to the categories where less money is gambled. As the daily amount of money wagered increases, the percentage of players progressively decreases. The maximum amount of money spent daily was also examined for each gender, and the results did not show statistically significant differences in the in-person modality [χ^2^ (4, 395) = 2.543, *p* > 0.05] or the online modality [χ^2^ (4, 156) = 2.139, *p* > 0.05]. However, a certain tendency can be observed where, in the categories in which larger amounts of money are bet daily, the percentages of boys are higher than those of girls.

We then examined the possible relationship between the maximum amount of money spent in a day and the age of the participants. The results of this analysis revealed no statistically significant differences between age groups in the categories of gambling intensity in the in-person modality [χ^2^ (16, 395) = 21.649, *p* > 0.05] or the online modality [χ^2^ (16, 156) = 20.53, *p* > 0.05]. 

### 3.2. Relationship between Gambling and Mental Health

Next, we analyzed the possible relationships between gambling and various mental health indicators. A MANOVA was performed, with the fixed factor being membership in a gambling group (online or in-person) in the past 12 months, and the dependent variables being the scores on emotional and behavioral problems, self-esteem, symptoms of depression, and subjective wellbeing. 

The Wilks λ value confirmed the existence of statistically significant differences (λ Wilks = 0.931, F = 16,295, *p* < 0.05, partial *η*^2^ = 0.069). Individual ANOVAs found statistically significant differences in almost all the dimensions analyzed (see Table 5). Only the variable showing the total SDQ problem score revealed no differences (*p* > 0.05).

## 4. Discussion

The present study aimed to analyze gambling prevalence, study its expression according to gender and age, and explore its possible relationship with indicators of mental health and personal wellbeing in a representative sample of adolescents in Spain. 

Regarding the first objective, the results indicate that 20.6% of the adolescents surveyed had gambled in the past 12 months. It is worrisome to note that this prevalence represents an increase with respect to the prevalence detected in the ESPAD report in 2015, when the percentage of Spanish adolescents who had gambled money in the past 12 months was 16% [2]. In fact, the data provided by the present study coincide with the increase observed (around 22%) in the latest report available at the European level [6].

The analysis of gambling frequency depending on its form showed higher frequencies for in-person gambling than for online gambling. Specifically, 24.9% of the sample reported having gambled in person in the past 12 months, compared to 10.2% who did so online. The gambling frequencies in this study are lower than those found in studies carried out with larger samples [18,30].

The study of gambling frequency according to its type yielded some very clear conclusions that agree with the results obtained in similar studies. In both in-person and online gambling, sports betting is the most prevalent type of gambling, with significantly higher frequencies than the other types of gambling. Similarly, Cámara et al. [31] state in their study that the most prominent type of gambling is sports betting. International studies have also detected this preference for sports betting [32]. In addition, the National Drug Plan [4] highlights video games as the most prevalent type of online gambling and lotteries as the most prevalent type of in-person gambling in 2018. It is evident, therefore, that sports have an influence on gambling games, either through sports betting or through football pools. Moreover, the increasing expansion of video games has reached playing for money, and betting money in video games is becoming increasingly prevalent in adolescents. It is important to note that the greater addictive potential of these types of gambling is related to pathological gambling, and so special attention should be paid to these types of games when carrying out prevention programs for adolescents.

The analysis of gambling intensity, measured by the maximum daily amount wagered in the past 12 months, concluded that most of the participants were in the lower intensity categories. In fact, 85.2% and 91.2% of the sample indicated that they had not gambled more than €30 in a day online or in person, respectively. However, as the intensity categories reach higher amounts of money, online gambling becomes more prevalent than in-person gambling. This situation shows that gamblers who bet higher daily amounts of money do so more frequently online. In this regard, it is interesting to highlight the relationship found by the Dirección General de Ordenación del Juego [33] between the average monthly expenditure on gambling games and the level of pathology of the gambler. The more pronounced the gambling problem, the greater the investment made by the player. Therefore, gambling prevention programs should focus on gamblers who bet larger amounts of money, due to their risk of suffering from problem gambling.

The second objective of the study was to analyze gambling frequencies as a function of gender and age. Regarding the gambling frequency level based on gender, the results support the existence of considerably higher frequencies in the male gender compared to the female gender, both in-person and in online gambling. This situation is frequent in most studies similar to the present one [34,35], and even in the National Drug Plan [4], whose 2018 results follow the same trend as those obtained in this study. Therefore, being a boy can be considered a risk factor for participating in gambling, and so it is necessary to adopt a gender perspective in the prevention and intervention of gambling behaviors.

In the analysis of gambling prevalence according to age, the results show that the prevalence grows as the age increases in both gambling modalities, with statistically significant differences found in in-person gambling from the age of 16. The National Drug Plan [4] yields results in its 2018 report that coincide with those found in this study, although it shows higher gambling prevalence rates. Cámara et al. [31] confirm the increase in the prevalence of gambling with age, and they establish 16 years old as the age when it begins to grow considerably. Likewise, not only does the gambling prevalence increase as the age increases, but also the frequency. Low gambling frequency is common in the very young. However, as adolescents get older, moderate and high frequencies become more prevalent and surpass the low frequency [34].

The age of initiation in gambling was also analyzed, and the results showed that 14 years old is the age when adolescents most often start gambling. This fact is alarming and consistent with what has been found in other studies, such as the one by Lamas et al. [36]. Therefore, it is necessary to develop prevention programs with groups that are at risk of beginning to gamble, in order to avoid the consequences this behavior can trigger. 

To address the third objective of the study, we analyzed the relationship between gambling and various mental health indicators. In the case of the SDQ scales, the results show that the participants who gambled during the past year obtained lower scores on emotional problems than those who did not gamble. This result is surprising because emotional problems have been linked to gambling in several studies [37], and particularly to pathological gamblers [38]. With regard to behavioral problems, gamblers who have bet money show higher scores. This finding confirms the results obtained by Paleologon et al. [39], who add that gamblers with gambling pathologies present particularly high scores on conduct problems. However, participants who claimed to have gambled money scored lower on problems with peers. In this regard, Welte et al. [40] state that when adolescents become initiated in gambling, they tend to socialize with peers who also gamble, which means that gambling is less perceived as a high-risk activity. As for hyperactivity difficulties, the highest scores correspond to those who had gambled money, and hyperactivity (and even inattention) has been related to gambling problems in numerous studies [39,41,42]. Likewise, it has been suggested that people with ADHD and people with gambling pathologies share similar behavioral regulation deficits [43]. The results of the analysis of prosocial behavior coincided with those of Paleologon et al. [39]), who stated that gamblers who had gambled money obtained lower scores than those who had not.

The analysis of the level of self-esteem as a function of having gambled money in the past 12 months yielded higher self-esteem scores in those who had gambled money. This result contrasts with those obtained in previous studies [10,38]. For example, Kaare et al. [38] found that gamblers with gambling pathologies reported significantly lower self-esteem than the control group. However, other studies point out that participants with moderate self-esteem have higher preferences for risky gambling than those with high and low self-esteem [44]. Therefore, although some authors consider low self-esteem to be a risk factor for gambling, others emphasize the need to pay more attention to the majority groups (moderate self-esteem), in order to prevent future gambling-related behaviors that may develop in adult life.

The depressive symptomatology of the sample was also analyzed, and the results show that participants who had gambled money in the past year had lower depression scores, in contrast to studies linking gambling in adolescents to depressive characteristics [14]. However, Moodie and Finnigan [45] note in their study that, although gamblers had higher depression scores than the control group, when only gamblers who did not seek treatment were included in the analyses, no differences in depression scores were found. In addition, some studies argue that depression precedes addiction, and that gambling is perceived as a solution to depressive states [46]. Therefore, if we take into account the influence of gambling on pre-existing emotional problems, it becomes crucial to identify the factors related to gambling disorders in adolescence, in order to carry out effective prevention and treatment.

Finally, after analyzing the subjective wellbeing of the sample, the results showed that those who had gambled money in the past year had higher scores on subjective wellbeing and life satisfaction. These results are surprising considering studies such as the one by Oei and Goh [47]), who show that having higher life satisfaction reduces the likelihood of experiencing gambling problems. However, the Directorate General for Gambling Regulation [33] examined the relationship between the degree of satisfaction with life and the degree of problem gambling, and one of the results pointed out that people who had gambled money but were not at risk of experiencing gambling problems had higher life satisfaction indices than people who had not gambled.

Taken together, this last group of results could support this hypothesis. Young participants who do not present emotional problems or depressive symptoms and have a good level of self-esteem and an adequate level of wellbeing may be using gambling for fun. They may be influenced by the advertising of an increasingly powerful industry, without suspecting that these behaviors could have consequences for their subsequent wellbeing. In fact, studies such as the one by Perez [48] state that the enjoyment produced by gambling is, along with the economic motivation, one of adolescents’ main reasons for participating in gambling. The same idea stems from Neighbors et al. [49], whose study with university students revealed that most participants gambled to win money and have fun.

This study has some limitations that should be considered when interpreting the results and relating them to future research. First, the conclusions obtained are conditioned by the self-report measurement instruments used. The prevalence of gambling and the indicators of mental health and personal wellbeing are based on self-report measures, which limits the ability to compare the results to those from other studies. Second, this study is solely based on quantitative methods, further studies should combine quantitative and qualitative research techniques in order to interpret the results in a richer and contextualized way. Third, the cross-sectional nature of this study limits the possibility of determining possible causal relationships between gambling and the variables explored; therefore, the conclusions drawn from the present study should be analyzed in longitudinal research in order to confirm them and study their tendency over time. Finally, despite the stratified random sampling by conglomerates, the sample is limited to Spanish adolescents from a particular autonomous community, which impacts the generalizability of the study’s findings. Given the peculiarities, diversity and plurality of the nation, future studies could examine the hypothesis in other regions. 

## 5. Conclusions

In carrying out this study, a network of information was obtained that allows a more or less real characterization of the current situation of gambling among adolescents in Spain. All this information should be used to design and implement prevention programs, with the aim of advancing education about gambling before the behavior develops. As we have seen, gambling involves a set of cognitive, behavioral, and social dimensions, and so all these variables should be considered when analyzing the behavior. Particularly, in this study, the results reveal the existence of positive and negative relationships between gambling and the different scales of mental health and personal wellbeing. Some of them may seem contradictory, but taking into account the existing consensus about the negative influence of gambling on mental health in adulthood, it can be assumed that in adolescence some psychological problems are not yet affected, and their relationship with gambling is still difficult to determine. Furthermore, this paper cites some studies that show relationships between some mental health indicators (emotional problems, behavioral problems, hyperactivity, or self-esteem difficulties) and gambling when gambling pathologies are present. It is possible that in the present study these relationships have not yet been established because they only appear when cases become pathological. A reasonable hypothesis is that the psychological impact depends on additional variables such as intensity, age of initiation, mode and motivation of initiation, type of game or the personal and contextual characteristics of the players. Future studies should address these questions to determine which profiles and which conditions are associated with an increased risk of pathological gambling and, consequently, merit a preventive, therapeutic or combined approach.

In any case, considering the increasing prevalence of gambling and the consequences linked to this behavior, the scarcity of prevention programs for adolescents is surprising. Hopefully, the present study will help to develop effective prevention initiatives. Overall, this study offers data indicating that there is a significant proportion of adolescents who have started gambling and an analysis that indicates relationships between gambling and some poorer mental health indicators. This requires that authorities take into account the need to include preventive actions that include gambling among potential risk factors for mental health. Undoubtedly, educational centers are a "natural" and ideal environment to develop and implement actions to promote emotional well-being. In this sense, a suitable perspective to carry it out may be the whole-school approach. This approach covers all aspects of the school experience, including the politics and culture of the school, classroom practice, as well as all the relationships that take place in the school (between students, teachers, and families). Likewise, this recognition obliges the public authorities to adopt measures aimed at its regulation and the design of public intervention policies aimed at young people, with special emphasis on the control of the influence of advertising, among others many other aspects.

## Figures and Tables

**Table 1:** Gambling prevalence in the past 12 months by gender and modality (N = 1790).

Gambling	In Person						Online						Total					
	M		F		Total		M		F		Total		M		F		Total	
	n	%	n	%	n	%	n	%	n	%	n	%	n	%	n	%	n	%
I didn’t gamble for money	494	60.5	838	87.2	1345	75.1	662	81.1	933	97.1	1608	89.8	533	65.3	878	91.4	1411	79.4
I gambled for money	332	39.5	123	12.8	445	24.9	154	18.9	28	2.9	182	10.2	283	34.7	83	8.6	366	20.6

*Note*. M = male; F = female.

**Table 2:** Prevalence of the frequency of the type of game according to the modality.

	Prevalence In-Person Gambling		Prevalence Online Gambling	
Types	n	%	n	%
Lotteries	58	7.4	22	7.1
Instant lotteries	43	5.5	17	5.5
Football pools	90	11.5	31	10
Sport bets	219	28	99	32
Betting on horse races	20	2.6	7	2.3
Slot machines	57	7.3	9	2.3
Card games with money	105	13.4	29	9.4
Bingo	88	11.2	4	1.3
Videogames	25	3.2	68	22
eSports or electronic sports	13	1.7	15	4.9
Casino games	28	3.6	6	2
Games in gambling halls	36	4.6	2	0.7

**Table 3:** Mean age of initiation in gambling depending on the modality and gender.

Mean	In-Person Game	Online Game
General	14.31	14.47
Male	14.39	14.48
Female	14.05	14.38

**Table 4:** Distribution of the maximum amount of money spent in one day according to gender and gambling modality (%).

	In-Person Gambling			Online Gambling		
Categories	Total	M	F	Total	M	F
Less than 6 euros	70.6	70.3	71.6	55.1	53.9	66.7
Between 6 and 30 euros	20.3	19.6	22	30.1	30.5	26.7
Between 31 and 60 euros	5.3	5.9	3.7	7.7	8.5	0.0
Between 61 and 300 euros	2.8	2.8	2.8	5.1	5.0	6.7
More than 300 euros	1	1.4	0.0	1.9	2.1	0.0

*Note*. M = Male; F = Female.

**Table 5:** Comparisons of mean scores on the variables according to whether they gambled money in the past 12 months.

	Gambled Money in the Past Year (n = 365)	Did not Gamble Money in the Past Year (n = 1416)			
	M (SD)	M (SD)	F	*p*	Partial *η*^2^
Emotional symptoms	2.85 (2.13)	3.60 (2.45)	28.265	0.000	0.016
Behavioral problems	2.20 (1.65)	1.61 (1.50)	43.052	0.000	0.024
Peer problems	1.26 (1.30)	1.50 (1.65)	6.274	0.012	0.004
Hyperactivity	4.79 (2.16)	4.24 (2.16)	19.069	0.000	0.011
Prosocial	8.23 (1.50)	8.65 (1.39)	24.791	0.000	0.014
Total difficulties	11.11 (4.64)	10.95 (5.23)	0.296	0.587	0.000
Self-esteem	32.17 (4.87)	30.46 (5.67)	27.962	0.000	0.015
Depression	15.96 (3.78)	16.53 (4.66)	4.688	0.031	0.003
Subjective well-being	7.97 (1.67)	7.70 (1.91)	6.095	0.014	0.003

*Note*. M = Mean; SD = Standard deviation.

## Data Availability

The data presented in this study are available on request from the corresponding author. The data are not publicly available due to the participants were minors.
